# Body size variation in aquatic consumers causes pervasive community effects, independent of mean body size

**DOI:** 10.1002/ece3.3511

**Published:** 2017-10-22

**Authors:** Bradley E. Carlson, Tracy Langkilde

**Affiliations:** ^1^ Department of Biology, Intercollege Graduate Degree Program in Ecology, and Center for Brain, Behavior, and Cognition The Pennsylvania State University University Park PA USA

**Keywords:** body size, intraspecific trait variation, size structure, tadpoles, zooplankton

## Abstract

Intraspecific phenotypic variation is a significant component of biodiversity. Body size, for example, is variable and critical for structuring communities. We need to understand how homogenous and variably sized populations differ in their ecological responses or effects if we are to have a robust understanding of communities. We manipulated body size variation in consumer (tadpole) populations in mesocosms (both with and without predators), keeping mean size and density of these consumers constant. Size‐variable consumer populations exhibited stronger antipredator responses (reduced activity), which had a cascading effect of increasing the biomass of the consumer's resources. Predators foraged less when consumers were variable in size, and this may have mediated the differential effects of predators on the community composition of alternative prey (zooplankton). All trophic levels responded to differences in consumer size variation, demonstrating that intrapopulation phenotypic variability can significantly alter interspecific ecological interactions. Furthermore, we identify a key mechanism (size thresholds for predation risk) that may mediate impacts of size variation in natural communities. Together, our results suggest that phenotypic variability plays a significant role in structuring ecological communities.

## 1 INTRODUCTION

Declines in biodiversity have led to considerable research on the consequences of species diversity for ecosystem processes (Hooper et al., 2005; Loreau et al., 2001; Srivastava & Vellend, 2005). While the study of “biodiversity–ecosystem function” relationships has yielded complex results and engendered some controversy (Loreau et al., 2001; Srivastava & Vellend, 2005), the general consensus is that ecosystem properties are altered by changes in species diversity, which are mediated by the influence of diversity on community structure and interactions (Balvanera et al., 2006; Hooper et al., 2005). This is thought to result from species differences in functional traits that mediate their ecological roles (Chapin et al., 1997; Norberg et al., 2001).

Ecologists typically focus on average (mean) values of traits within species when characterizing functional trait diversity (Cianciaruso, Batalha, Gaston, & Petchey, 2009; Fritschie & Olden, 2016). However, within a single species, individuals exhibit significant variability in ecologically relevant phenotypic traits. This intraspecific trait variation is a component of the total functional diversity in a community (Albert et al., 2010; Violle et al., 2012), and initial research suggests that the degree of variance around mean trait values of a species can have strong impacts on the response of that species to its environment and, consequently, on the community with which it interacts (Bolnick et al., 2011). Populations with more variable traits may interact differently with the biotic and abiotic environment than do more homogenous populations, either immediately (e.g., Crutsinger et al., 2006) or over several generations (e.g., by permitting alternative evolutionary or population dynamics that impact the community; Becks, Ellner, Jones, & Hairston, 2010). Naturally occurring phenotypic or genetic variants within a species can have differential ecological impacts, as demonstrated by studies of trait differences between populations (Bassar et al., 2010; Palkovacs & Post, 2009) or between age classes within populations (Miller & Rudolf, 2011; Rudolf & Rasmussen, 2013a), and comparisons of populations of asexual organisms that differ in genotypic diversity (Hughes, Inouye, Johnson, Underwood, & Vellend, 2008). This indicates that researchers should investigate the contribution of diversity *within* species as well as among species to fully understand the ecological significance of biodiversity for communities and, thus, ecosystems (Rudolf & Rasmussen, 2013b; Violle et al., 2012).

Size—in particular, body mass—is considered both one of the most variable and most ecologically significant traits (Woodward et al., 2005) and is therefore an important characteristic for studying the ecological consequences of intraspecific trait variation. Within species, size usually varies across ontogeny (Polis, 1984; Rudolf & Rasmussen, 2013b), but can also vary within same‐aged cohorts due to stochastic (e.g., environmental) or deterministic (e.g., genetic) variation in factors that impact growth rate (Pfister & Stevens, 2002). Resource limitation at high population density frequently generates higher size variation (Uchmański, 1985), as can other ecological factors such as predation risk (Peacor, Schiesari, & Werner, 2007), resulting in predictable patterns of size variation across environments. Size also plays a central role in determining basic ecological properties of individuals (Woodward et al., 2005), such as metabolic rate (Brown, Gillooly, Allen, Savage, & West, 2004) and susceptibility to predators (Cohen, Pimm, Yodzis, & Saldana, 1993). The mean body size of different species may be a central component of functional diversity (Woodward et al., 2005) and therefore an important element in the study of relationships between biodiversity and ecosystems (Reiss, Bailey, Perkins, Pluchinotta, & Woodward, 2011). Furthermore, the size variation that occurs within a single species can be substantial enough that resource use of different size classes may vary as much as between different species (Rudolf & Rasmussen, 2013b). This may result in populations that contain individuals of the full spectrum of sizes having an overall greater niche width than do populations of similar‐sized individuals (Polis, 1984), which can significantly alter population and community dynamics (De Roos, Persson, & McCauley, 2003).

A number of studies have demonstrated that variably sized (or size‐structured) populations produce different ecological consequences than do homogeneous populations (e.g., Asquith & Vonesh, 2012; Fritschie & Olden, 2016; Kishida, Mizuta, & Nishimura, 2006; Peacor & Werner, 2001; Rudolf & Rasmussen, 2013a; Yamaguchi & Kishida, 2016). Such studies have tended to focus on distinct age classes, such as cohorts from different breeding events (e.g., Peacor & Werner, 2001). Understanding the effects of the more subtle and continuous variation within individual age classes that is ubiquitous in natural populations (e.g., Ingram, Stutz, & Bolnick, 2011) is necessary to expand and refine our understanding of the ecological consequences of size variation. Similarly, studies that manipulate variation in body size generally simultaneously alter mean body size, population density, or total biomass (a composite of the two former population characteristics), and observational studies cannot easily disentangle these factors (Fritschie & Olden, 2016).

Avoiding these potentially confounding effects and thus isolating the direct consequences of variance around the mean body size requires experiments that manipulate variation in size while holding mean size and population density constant. Such studies are rare (but see Ingram et al., 2011). Moreover, examining the multitrophic level impacts of size structure (e.g., Rudolf & Rasmussen, 2013a) furthers our current understanding of the effects of body size variation, which predominately focuses on implications for the manipulated species and/or a single interacting species (e.g., Asquith & Vonesh, 2012). Size structure within a single species may influence its own resources/prey, its predators/parasites, and, indirectly, other species with which the immediately lower or higher trophic levels are interacting; however, this is rarely evaluated.

We experimentally test whether the extent of continuous variation in size (in this case, body mass) in consumer populations influences their interactions with both lower and higher trophic levels, while holding mean body size and population density constant. We performed this experiment using wood frog tadpoles (*Lithobates sylvaticus*), their food resources (periphyton), their predators (newts), and a third group which may act as an alternative prey for these predators and potentially a competitor/prey for the tadpoles (microcrustacean zooplankton). Wood frog tadpoles are a common amphibian consumer in forest ponds throughout much of eastern and northern North America, and they can exhibit substantial size variation in same‐aged cohorts, especially under strong intraspecific competition (Peacor & Pfister, 2006). We created matched populations of tadpoles of the same mean mass and abundance that had either low or high variance in mass around that mean. Our first prediction is that variably sized populations of tadpoles should have different effects on food resource biomass. Food intake, among other ecological properties, often scales nonlinearly with body size. Due to Jensen's inequality (a mathematical property of nonlinear functions), the total food intake in a size‐variable population should be higher or lower (depending on whether the function is convex or concave, respectively) than a homogenous population (Bolnick et al., 2011; Ruel & Ayres, 1999). Food intake of tadpoles exhibits a nonlinear, concave relationship with mass (Werner, 1994). Therefore, variably sized populations should consume less of the biomass of their resources, including periphyton (biofilms of bacteria, algae, and fungi) and detritus (e.g., leaf litter), although they can also consume substantial amounts of animal matter such as microcrustacean zooplankton (Schiesari, Werner, & Kling, 2009).

We crossed the high versus low size variation treatment with the presence or absence of newt predators. A more variably sized tadpole population should affect predators and alternative prey differently than a uniformly sized population. Eastern newts (*Notophthalmus viridescens*) are aquatic salamanders that are common predators of wood frog tadpoles as well as zooplankton and insects (Burton, 1977; Petranka, 1998). Like many predators, these newts are gape‐limited (Urban, 2008), and wood frog tadpoles grow too large for newts to consume midway through development (at approximately 300–400 mg; Carlson, personal observations). A population composed of similarly sized tadpoles will synchronously reach this size threshold for predation risk, and the majority of the population will become invulnerable to predators, while a population of the same average size that includes both large and small individuals will contain a smaller percentage of individuals that are vulnerable to predation but over a longer period of time as smaller tadpoles enter this size range. Consequently, we expect that highly size‐variable groups of tadpoles that contain more vulnerable individuals would exhibit reduced survival in the presence of predators, due to the constant presence of predator‐vulnerable individuals. (If the mean body size were below this threshold, however, increased size variation would lead to more tadpoles that are not at risk of predation.) Furthermore, many consumers (including the tadpoles in this study) decrease foraging activity under predation risk (Lima & Dill, 1990; Van Buskirk & Yurewicz, 1998), a behavioral response that lessens as tadpoles grow larger and less vulnerable (Puttlitz, Chivers, Kiesecker, & Blaustein, 1999). The presence of small, vulnerable consumers in a variable population would therefore result in reduced foraging and greater biomass of food resources via a density‐mediated and/or behaviorally mediated trophic cascade (assuming larger prey individuals do not exhibit a compensatory increase in foraging in response to relaxed competition).

We also anticipate that the greater availability of vulnerable prey within a size‐variable tadpole population would result in greater growth of the newts and a reduced reliance on alternative prey (provided the tadpoles are preferred and of greater profitability than alternative prey). Among the prominent prey resources in most ponds—and those measured in this study—are the microcrustacean zooplankton, particularly cladocerans (or “water fleas”) and copepods. The substantially larger size of the tadpoles compared to these zooplankton suggests that they are a more profitable food source, which is supported by the observation that newts from ponds with dense wood frog tadpole populations tend to have greater mass and body condition (Carlson & Langkilde, 2016). Therefore, the variably sized tadpole populations should lead to an increase in the abundance of zooplankton when newts are present. In some systems, however, the enhanced growth of predators may enhance their capacity to feed upon alternative prey, resulting in greater predation pressure (Takatsu & Kishida, 2015); a form of apparent competition (Holt, 1977), although in our study, there are no alternative prey that are larger than the tadpoles. Finally, changes in tadpole abundance and foraging may also indirectly influence zooplankton populations. Cladocerans are largely indiscriminate filter feeders upon phytoplankton and bacteria, while copepods selectively prey upon larger phytoplankton and protozoans (Sommer & Sommer, 2006). Tadpole consumption of periphyton and excretion of sequestered nutrients into the water column may increase the availability of phytoplankton and other microbes, supporting the microcrustacean populations (Wilbur, 1997).

Overall, we found that altering the extent of size variation in the tadpoles ultimately affected every trophic level examined, and these effects usually interacted with the presence of predators, suggesting that size‐dependent predation risk—rather than Jensen's inequality—mediated many of these impacts.

## 2 MATERIALS AND METHODS

### 2.1 Study subjects

*Lithobates sylvaticus* eggs were collected between 15 April 2013 and 22 April 2013, from a pond in State Game Lands #176, Centre County, PA, USA (40.7649 N, 78.0163 W). We could not determine exactly how many sibling groups were represented as *L. sylvaticus* oviposit egg masses communally and after about a week it is difficult to distinguish individual clutches, but we estimate that eggs from 15 to 25 clutches were collected. Eggs were hatched over a period of approximately 2 weeks and tadpoles initially reared outdoors in either 9‐L plastic tubs (single clutches, where these could be distinguished) or 100‐L plastic wading pools (multiple clutches), both covered in 60% shade cloth. All tadpoles were then divided approximately evenly between three outdoor cattle tank mesocosms (hereafter, “holding tanks”). Each holding tank held approximately 800 L of well water, 200 g (after air‐drying) of deciduous leaf litter (primarily *Quercus velutina* and *Q. prinus*), and 12.5 g of rabbit chow as an initial food source. Prior to introducing tadpoles, we added a 1‐L inoculate of water from another *L. sylvaticus* pond to introduce natural microbes, algae, and plankton. We did not count the tadpoles added to the holding tanks, but estimate that about 1,500–2,000 tadpoles were in each tank. This density was intended to produce high levels of competition needed to produce the size variation to establish our experimental groups of tadpoles (Peacor & Pfister, 2006), while being within the range of densities observed in the field (Biesterfeldt, Petranka, & Sherbondy, 1993; Carlson, personal observations).

### 2.2 Experimental setup

We conducted a 2 × 2 factorial experiment in which we created groups of tadpoles that differed in the extent of size variation (“high” or “low”) and manipulated the presence of a newt predator (“predator” or “no predator”) in pond mesocosms. We placed 36 1100‐L round plastic cattle tanks (“mesocosms”) in a 9 × 4 grid in an open field, with one replicate (mesocosm) of all four treatment combinations in each of the nine blocks (total *N* = 36). All experimental procedures were performed identically within each block. Blocks differed in spatial location, the dates of setup and data collection, the mean mass of tadpoles, and the size distributions (see below); and these differences were accounted for in analyses by incorporating block effects. Mesocosms were filled with approximately 600 L of well water and provided with leaf litter, rabbit chow, and 1‐L inoculates of pond water as described above for the holding tanks, 21–27 days before adding tadpoles. To ensure the development of a robust zooplankton community, the pond water from which inoculates were drawn received additional zooplankton collected with a fine mesh net, and this pond water was then well‐mixed between each inoculation to ensure similar initial populations of zooplankton. Three 15 × 15 cm gray ceramic tiles were leaned upright against the eastern side of the mesocosm wall to provide a surface for periphyton growth for subsequent measurement (Relyea, 2005). Mesocosms were covered with 60% shade cloth to limit direct sunlight and prevent colonization by unwanted animals. During periods when the lids were off (during behavioral observations and sampling), however, insects did colonize the mesocosms. Chironomid midge (Diptera: Chironomidae) larvae and/or the tubes they characteristically construct and inhabit were present in all of the mesocosms, and other aquatic insects (e.g., Hemiptera: Gerridae, Coleoptera: Hydrophilidae) were also observed in lower abundance at the conclusion of the experiment in many mesocosms. These and other sources of community variation among tanks certainly contribute to differences in mesocosms at the conclusion of the experiment; however, treatments were randomly assigned, and therefore, this variation in initial community structure and composition is accounted for during statistical analysis.

**Figure 1:**
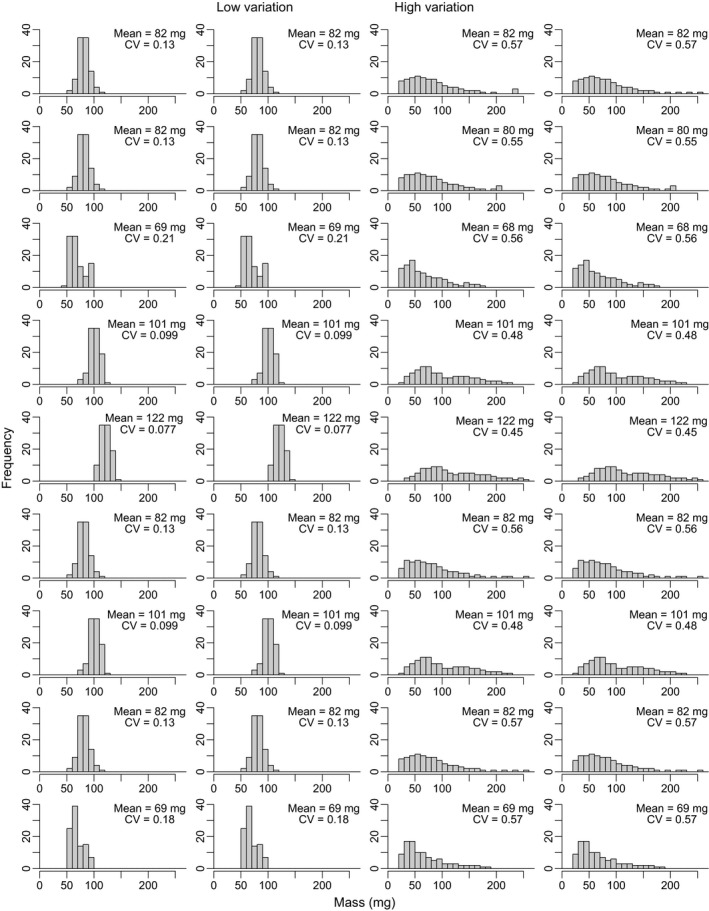
Initial size distributions for low and high size variation treatment groups of tadpoles. Rows represent blocks of four mesocosms (two of each size variation treatment per block). Within blocks, mesocosms are matched for the same mean body mass and were set up and measured on the same dates. For each mesocosm, the mean body mass and the coefficient of variation (CV) are listed

Over a 7‐day period, we added 100 tadpoles to each tank, with all tadpoles in a given tank introduced on the same day. We collected tadpoles haphazardly from the holding tanks and weighed them individually to the nearest milligram, after wicking away excess water with a paper towel. We sorted tadpoles by mass by temporarily holding them in 250‐ml containers corresponding to 10‐mg range size classes (e.g., 90–100 mg). We then stocked mesocosms with tadpoles from these containers when enough had been weighed in every required size class to stock an entire block of mesocosms. For the high variation treatment, our aim was to approximate the degree of variability that arises naturally in this species at high densities in the field (Carlson, unpublished data) and in experimental mesocosms (Peacor & Pfister, 2006). Size distributions were therefore developed to create coefficients of variation (CVs) of 0.45–0.57 in the high variation treatment group. For the low variation treatment, we only chose tadpoles within a narrow size range, producing CVs of 0.08–0.21. Importantly, the average mass (and hence the total biomass) was the same for both high and low variation treatments in a given block, and the density/abundance was the same (Figure 1). The mean mass did however differ (68–122 mg) among blocks; this variation in mean mass among blocks seems to have been transitory, as there was no correlation between initial and final mean mass in each mesocosm at the conclusion of the experiment (*r* = .03, *t*_34_ = 0.19, *p* = .85) and no variation among blocks in final mean mass (ANOVA: *F*_8,27_ = 0.62, *p* = .75). Given the sizes of the tadpoles available, it was not possible to establish symmetrical, normal distributions, although the distributions were approximately log‐normal. Furthermore, the range of sizes present differed along with the variance in the two treatments. Although we did not explicitly seek to evaluate it in this study, it is possible that individuals in certain sizes act as keystone members of the population and that their presence alone is more ecologically significant than the variance in the population. However, the inclusion of a particularly important size class is a consequence of a population that is highly variable in size (similar to the sampling effect in species richness–ecosystem function relationships; Fridley 1999), and we therefore consider this a potential mechanism for the effects of size variation rather than an alternative to size variation as the driver. The consequences of size variation, although, would depend on which specific size classes occur in different groups, which we are not able to address in this study. For practical reasons, we could not determine the age (since hatching) or developmental stage (Gosner, 1960) of each tadpole and therefore cannot be confident that these were also similar between treatments. We note that all tadpoles were freely swimming and feeding without external gills (and thus at least stage 25) and had little or no development of the hindlimbs (approximately stage 34 or earlier) and were consequently similar in morphology aside from size differences.

Two male newts were added to each mesocosm receiving the predator treatment (2 mesocosms per block). We collected 36 newts by dipnet from a single large pond in Pennsylvania State Game Lands #176. Wood frogs are not known to regularly breed in this source pond, although their tadpoles are found in other nearby ponds along with newts, and tadpoles of congeneric green frogs are found in this pond. Newts were selected to all be similarly sized adults, although mass varied from 1.53 to 3.04 g. Visual assessment suggested most of this variation in mass was due to variation in body condition (or the recent consumption of a large meal) rather than overall size, which would affect gape width. These newts were temporarily housed in a tadpole‐free mesocosm, from which they were haphazardly collected and introduced to mesocosms on the same day tadpoles were added. We first weighed each newt and noted its individually unique spotting pattern to permit us to document changes in mass throughout the study.

### 2.3 Behavioral observations

Beginning 10 days after adding tadpoles to a mesocosm, we observed tadpole and (in predator‐treated tanks) newt behavior once per day for 6 days. Inclement weather occasionally prevented data collection, and these six observation days were thus conducted over 6–8 consecutive days. Prior to behavioral observations, we removed mesocosm lids and waited at least 15 min for normal activity to resume. A single investigator (B. E. Carlson) then walked around each mesocosm and counted the number of tadpoles visible or moving. In statistical analyses, tadpole visibility was calculated as the proportion of tadpoles known to be alive at the conclusion of the experiment that were visible during the behavioral observations. This allows us to determine whether the predators may be inducing a hiding response (e.g., tadpoles beneath leaf litter; McIntyre, Baldwin, & Flecker, 2004). Activity rates were limited to the proportion of visible tadpoles that were moving, as we could not determine whether unobserved tadpoles were active. After the entire area of the mesocosm was surveyed for tadpoles, the observer (BEC) then began behavioral observations of newts (if present). He walked around each mesocosm slowly twice, while thoroughly visually searching for the newts. When a newt was found, he watched it for 120 s or as long as it remained visible. If one or both of the newts could not be located, they were documented as not visible; in most cases, they were probably hidden under leaf litter. During observations of visible newts, he counted (1) the number of movements, as they typically moved with repeated short swimming bouts and (2) the number of feeding strikes, characterized by a rapid lunge forward with a sudden stop and snapping of the mouth. Some of these strikes were clearly directed toward tadpoles, whereas in other cases toward prey not visible to the observer (presumably crustacean zooplankton or small insects).

### 2.4 Community responses

At the completion of the experiment, we collected data on periphyton biomass, zooplankton abundance, and newt mass. To measure periphyton biomass, two tiles were removed 16–18 days after adding tadpoles and stored at −20°C in plastic bags prior to analysis. We then thawed the tiles and removed all material from the exposed surface using distilled water and a scrub brush. The periphyton (suspended in water) was vacuum‐filtered through predried and preweighed Whatman GF/C glass microfiber filter paper. The filter paper was dried again, weighed to the nearest 0.1 mg, and the difference from the initial mass was taken as the periphyton biomass. Zooplankton samples were collected by dropping a 1.3‐cm‐diameter pipe into the water column at four standardized locations and collecting 30 ml of water each time (Relyea, 2005). The water was filtered through 80‐μm Nitex mesh and zooplankton preserved in 70% ethanol. Using a dissecting microscope, preserved zooplankton were counted and identified as daphniid cladocerans, nondaphniid cladocerans (primarily Bosminidae and Chydoridae), calanoid copepods, or cyclopoid copepods. Newts were weighed 21 days after introducing them to mesocosms to quantify changes in mass.

After 21 days, we collected all tadpoles from mesocosms, euthanized them with MS‐222, and preserved them in 70% ethanol. Preserved tadpoles were used to document survival rates, Gosner developmental stage (Gosner, 1960), and mass. The mass of alcohol‐preserved tadpoles likely deviates from their mass in life but should still provide a measure of relative differences in mass of individual tadpoles.

### 2.5 Statistical analysis

Full details of the statistical analysis are presented in Appendix S1. For most response variables, our general analytical approach was to fit either mixed effects ANOVAs (for normally distributed responses) or generalized linear mixed models (GLMMs, for non‐normal distributions), with fixed effects of treatments (low/high tadpole size variation and newt presence/absence) and their interaction and random effects of mesocosm (when repeated observations were made on a single mesocosm) and block. Exceptions to this approach include newt response variables (for which the effect of newt presence could not be considered) and the overall composition of the microcrustacean community (tested first as a MANOVA before conducting univariate analyses). Count variables (abundances of zooplankton, newt movements, and strikes) were fit to quasi‐Poisson distributions (accounting for overdispersion), and proportional variables (tadpole survival, visibility, and activity rates) were fit as quasi‐binomial distributions (Bolker et al., 2009). We also explored the contribution of differences between mesocosms in mean tadpole size and stage by evaluating models including these as covariates, because these could not be perfectly controlled independently of treatments. For responses associated with periphyton, zooplankton, and newts, we also included tadpole survival and behavior as covariates to preliminarily assess their role in mediating treatment effects on the community.

All analyses were conducted in R v3.1.0. We used the function *glmmPQL* in the package “MASS” to fit GLMMs. ANOVAs and MANOVAs were fit using functions in the base package. We evaluated normality of residuals, homogeneity of variance, and the presence of overdispersion, as appropriate, to validate the use of selected statistical models. We used α = 0.05 significance level, except for final mean mass of tadpoles (α = 0.025; see Appendix S1). Due to the large number of statistical tests involved in this study, there is an increased risk of generating “false positives” (significant results due to chance). This is an inherent statistical limitation for community and ecosystem‐level research in which the number of potentially important variables is high, while the capacity for replication is limited, impeding the ability to detect biologically significant effects (Moran, 2003). To provide balance between minimizing false positives and maximizing power, and to provide full transparency, we report and discuss the uncorrected *p*‐values while noting in the tables and figures which results remained significant after correcting *p*‐values for multiple testing by controlling for the false discovery rate (FDR; García, 2004). We applied this procedure to all *p*‐values generated without including covariates in the analysis (i.e., those that appear in Tables 1 and 2 and in Figure 4). We suggest that specific results that are not significant when correcting for FDR be approached cautiously and considered exploratory rather than confirmatory. Data are available from the Dryad Digital Repository (https://doi.org/10.5061/dryad.59j4m).

**Table 1:** Effects of size variation and newt presence on means and coefficients of variation (CV) of tadpole mass and Gosner stage (a–d), tadpole survival (e), visibility (f), and activity rates (g)

Response	Treatments/covariates	Test statistics	Significance
(a) Mean tadpole mass	Size variation	*F*_1,24_ = 0.78	*p* = .39
	Newt present	*F*_1,24_ = 14.6	***p*** ** = .0008** a
	Size × Newt	*F*_1,24_ = 2.07	*p* = .16
(b) Tadpole mass CV	Size variation	*F*_1,24_ = 60.1	***p*** ** < .0001** a
	Newt present	*F*_1,24_ = 0.07	*p* = .79
	Size × Newt	*F*_1,24_ = 0.42	*p* = .52
(c) Mean tadpole stage	Size variation	*F*_1,24_ = 5.67	***p*** ** = .03**
	Newt present	*F*_1,24_ = 1.12	*p* = .30
	Size × Newt	*F*_1,24_ = 1.75	*p* = .20
(d) Tadpole stage CV	Size variation	*F*_1,24_ = 49.7	***p*** ** < .0001** a
	Newt present	*F*_1,24_ = 0.12	*p* = .73
	Size × Newt	*F*_1,24_ = 0.12	*p* = .73
(e) Proportion surviving	Size variation	*t*_24_ = 0.56	*p* = .58
	Newt present	*t*_24_ = 5.59	***p*** ** < .0001** a
	Size × Newt	*t*_24_ = 0.30	*p* = .77
(f) Proportion visible	Size variation	*t*_24_ = −0.13	*p* = .90
	Newt present	*t*_24_ = −1.44	*p* = .16
	Size × Newt	*t*_24_ = −0.47	*p* = .64
(g) Proportion active	Size variation	*t*_24_ = 0.89	*p* = .38
	Newt present	*t*_24_ = 1.58	*p* = .13
	Size × Newt	*t*_24_ = −2.63	***p*** ** = .01**

All models were fit using ANOVA models with a random block effect (a–d) or generalized linear mixed models (GLMM) with quasi‐binomial distributions and random intercepts for block (e–g) and mesocosm (f–g). Bold *p*‐values are significant prior to correcting for false discovery rate

a Indicates results that remain significant (*p* < .05) after correcting for false discovery rate.

**Table 2:** Effects of size variation and newt presence treatments on periphyton biomass (log‐transformed) and microcrustacean zooplankton abundance

Response	Treatments/covariates	Test statistics	Significance
(a) Periphyton biomass	Size variation	*F*_1,23_ = 2.42	*p* = .14
	Newt present	*F*_1,23_ = 0.13	*p* = .72
	Size × Newt	*F*_1,23_ = 4.26	***p*** ** = .051**
(b) Microcrustacean community	Size variation	Pillai = 0.03 (*F*_4,21_ = 0.17)	*p* = .95
	Newt present	Pillai = 0.38 (*F*_4,21_ = 3.28)	***p*** ** = .03**
	Size × Newt	Pillai = 0.55 (*F*_4,21_ = 6.42)	***p*** ** = .002** a
(c) Total microcrustacean abundance	Size variation	*t*_24_ = 0.02	*p* = .99
	Newt present	*t*_24_ = −1.71	*p* = .10
	Size × Newt	*t*_24_ = 0.5	*p* = .62
(d) Daphniid cladocerans	Size variation	*t*_24_ = 0.92	*p* = .37
	Newt present	*t*_24_ = −1.86	*p* = .08
	Size × Newt	*t*_24_ = −0.71	*p* = .48
(e) Nondaphniid cladocerans	Size variation	*t*_24_ = −1.48	*p* = .15
	Newt present	*t*_24_ = −0.37	*p* = .71
	Size × Newt	*t*_24_ = −2.63	*p* = .08
(f) Calanoid copepods	Size variation	*t*_24_ = −2.09	***p*** ** = .05**
	Newt present	*t*_24_ = −3.07	***p*** ** = .005** a
	Size × Newt	*t*_24_ = 3.34	***p*** ** = .003** a
(g) Cyclopoid copepods	Size variation	*t*_24_ = 1.14	*p* = .26
	Newt present	*t*_24_ = 1.05	*p* = .30
	Size × Newt	*t*_24_ = −0.81	*p* = .43

All models were fit as linear mixed models (a), MANOVA (b) or quasi‐Poisson‐distributed GLMMs (c–g) with random intercepts for block. Bold p‐values are significant prior to correcting for false discovery rate

a Indicates results that remain significant (*p* < .05) after correcting for false discovery rate.

## 3 RESULTS

Differences in variation of tadpole mass (coefficients of variation; CV) between size variation treatments (low vs. high tadpole size variation) established at the start of this study were generally still present at the end of the experiment but were reduced (Table 1; Appendix S1; Figure S1). In only one block, the CV of mesocosms in the two size variation treatments overlapped. Furthermore, high variation in size also yielded high variation in developmental stage at the end of the experiment (Table 1; Appendix S1; Figure S1). Mean tadpole mass remained similar in the two size variation treatments at the end of the experiment (Table 1; Appendix S1; Figure S1) whereas, tadpole stage was higher on average in the low size variation treatment, although still within approximately 1 Gosner stage of that in the high size variation treatment (Table 1; Appendix S1; Figure S1).

**Figure 2:**
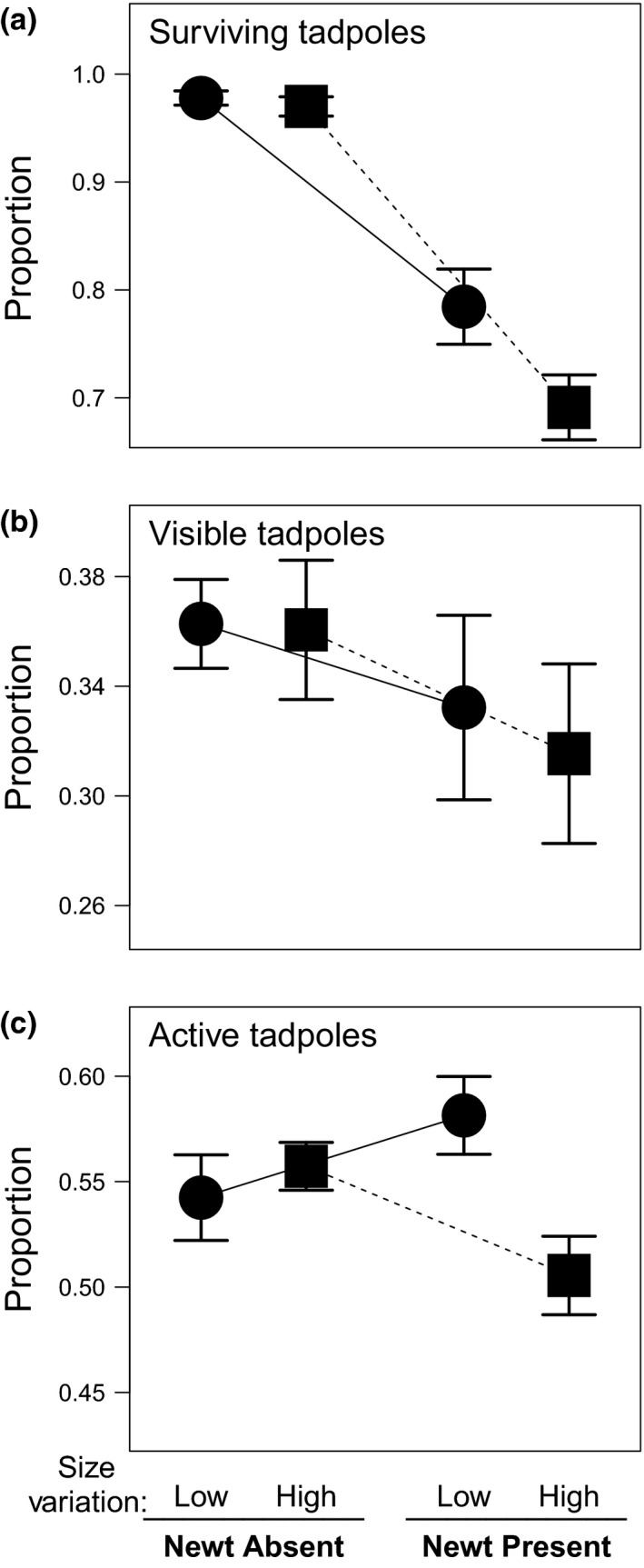
Treatment effects of tadpole size variation and newt presence on (a) tadpole survival, (b) proportion of tadpoles visible, and (c) proportion of visible tadpoles that were active. Values are mesocosm means ± 1 *SE*

Mean tadpole mass was significantly increased by the presence of newts, but tadpole developmental stage was not affected by newts. Newt presence reduced tadpole survival (Figure 2a; Table 1). Survival was not affected by size variation treatments or the interaction of size variation with newt presence (Figure 2a; Table 1). However, this may be a statistical artifact: Few tadpoles died when newts were absent—97% survival for high variation tadpoles and 97.8% survival for low variation tadpoles. Despite this small difference, high variation tadpoles were ~34% more likely to die. This mirrored the proportional difference in survival (39%) under newt predation (high variation tadpoles: 69.1%, low variation tadpoles: 78.4%), yielding no significant interaction between newt presence and size variation. The lack of a significant interaction therefore reflects the fact that risk of mortality with predators did not multiplicatively affect the increased risk of mortality already experienced by high variation groups of tadpoles. Treating the four treatment combinations as levels of a variable rather than as two interacting variables suggests there may be differences in survival between high and low variation tadpoles in the presence of newts (*t*_24_ = 2.41, *p* = .024) but not in the absence of newts (*t*_24_ = 0.56, *p* = .58), and thus, we consider this finding to be presently equivocal.

Tadpole visibility in mesocosms was not affected by either treatment or their interaction (Figure 2b; Table 1; Appendix S1; Table S1). In the presence of predators, a smaller proportion of visible tadpoles were active in the high size variation compared to the low size variation populations; however, there was no effect of size variation on activity when newts were absent (size variation treatment × newt presence interaction; Figure 2c; Table 1). This effect remained significant when accounting for differences in tadpole size and stage (Appendix S1; Table S1).

**Figure 3:**
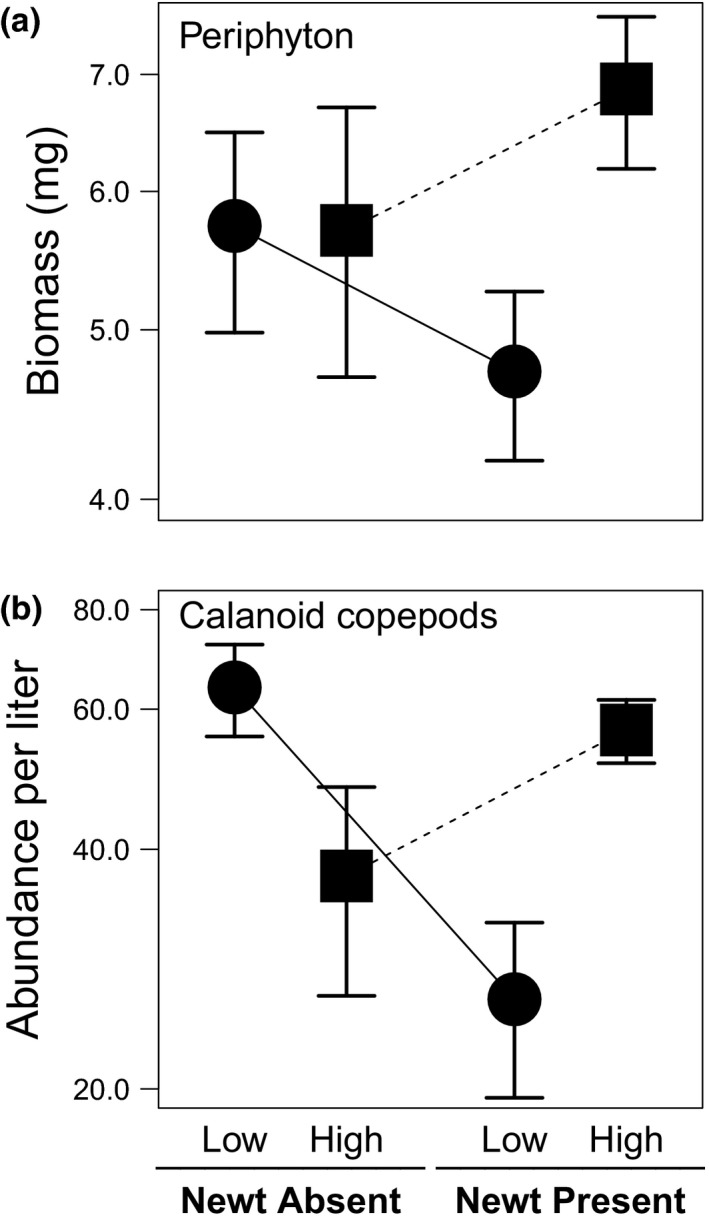
Treatment effects of tadpole size variation and newt presence on (a) periphyton biomass and (b) calanoid copepod abundance. Values are mesocosm means ± 1 *SE*. *Y*‐axes are presented on a logarithmic scale

Periphyton biomass was greater in the high size variation than low variation mesocosms, in the presence, but not in the absence, of newts (Figure 3a; Table 2). This effect persisted after accounting for other covariates (Appendix S1; Table S1). The microcrustacean zooplankton community was affected overall by an interaction between the size variation and newt presence treatments (Table 2). Univariate analyses of individual groups of zooplankton revealed that in the absence of newts, calanoid copepods were *more* abundant in mesocosms containing low variation groups of tadpoles than in those containing high variation groups; whereas in the presence of newts, they were *less* abundant in low variation than the high variation treatment (Figure 3b; Table 2). These effects on calanoid copepods were not significant when accounting for covariates for tadpole size, stage, behavior, and survival. In contrast, when including only tadpole covariates, nondaphniid cladocerans were significantly less abundant in mesocosms with high variation tadpoles when newts were absent, but tadpole size variation had the opposite effect when newts were present (Appendix S1; Table S2; Figure S2c). All other responses of zooplankton to treatments were nonsignificant (Table 1; Appendix S1; Table S2; Figure S2a,b,d).

**Figure 4:**
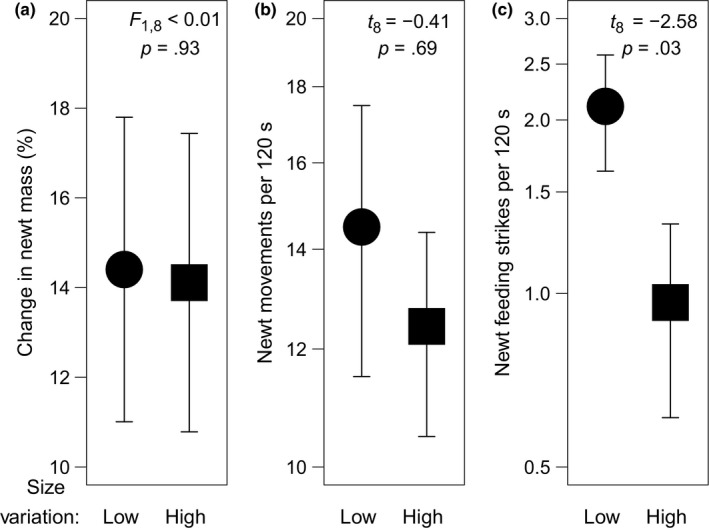
Treatment effects of tadpole size variation on (a) change in newt mass, (b) number of newt movements, and (c) number of feeding strikes by newts (during observation periods for b and c). Values are mesocosm means ± 1 *SE*, and *y*‐axes in (b) and (c) are presented on a logarithmic scale. None of the results depicted here remained significant (*p* < .05) after correcting for false discovery rate

Newt mass increased over the course of the experiment, and this weight gain was similar in both tadpole size variation treatments (Figure 4a). The number of times the newts moved during the observation periods did not differ between size variation treatments (Figure 4b), although there was a marginally nonsignificant decrease in movement rates of newts in high variation mesocosms when accounting for other covariates (*p* = .06; Appendix S1; Table S3). The number of feeding strikes by newts was significantly lower in mesocosms containing high size variation tadpoles than in those containing tadpoles of similar sizes (Figure 4c). This effect remained significant after accounting for potential mediating covariates (Appendix S1; Table S3).

## 4 DISCUSSION

We found that variation in body mass among individuals within a population appears to be an important determinant of ecological interactions in this system, demonstrating that intraspecific trait variation may have significant impacts on communities. Size variation within consumer populations had several ecological consequences, usually interacting with the presence of predators. Tadpole (consumer) populations with high size variation exhibited lower activity levels in the presence of newts (predators), consistent with an antipredator response (reduced activity to minimize detection by/encounters with predators; Relyea, 2001), whereas low variation groups did not exhibit antipredator behavior, likely due to reduced predation risk. Similarly, tadpole resources (periphyton) increased in the presence of newts in mesocosms containing highly size‐variable, but not similar‐sized, tadpoles. Microcrustacean communities (an alternative prey for the newts) were also altered by the interaction between tadpole size variation and the presence of their predator: Low size variation tadpole populations had higher calanoid copepod abundance in mesocosms without newts, while the opposite was true in mesocosms with newts. Finally, newts exhibited greater foraging effort (strikes at prey) in low variation groups of prey but gained weight at similar rates, suggesting lower availability of high‐quality food (tadpoles small enough for newts to eat) caused a compensatory increase in foraging on alternative prey (zooplankton).

We were unable to measure or control all potentially important characteristics of the mesocosm communities prior to introducing the tadpoles, and so it is possible that initially varying conditions could have contributed to these results. However, we are assuming that any variation would be random with respect to treatment and that our documented treatment effects are robust. So, why did size variation in tadpoles have such a broad impact on the mesocosm community in this study? We anticipated that ecological effects of size variation could be produced by size‐dependent predation risk and nonlinear scaling of food intake in tadpoles. We found no support for the latter, as size variation of tadpoles did not affect resource levels (periphyton) in the absence of predators, as would be predicted if nonlinear scaling of feeding was important. We did, however, find that the scaling of predation risk with body size appears to have been an important driver of community‐level impacts of size variation. As gape‐limited predators, newts are unable to feed upon prey that surpass a size threshold (Urban, 2008). (Alternatively, increased handling time of large prey may limit their profitability; Thompson 1975.) The amount of variance in tadpole size thus determines what proportion of the prey population falls above or below this threshold, and tadpoles on different sides of the threshold do not experience equivalent risk per unit mass. Consequently, the mean size of the population is insufficient for characterizing this interaction. In this study, the average size of tadpoles at the conclusion of the experiment (~640 mg) was larger than the size threshold at which wood frog tadpoles are rarely depredated by newts (approximately 300–400 mg; Carlson, unpublished data). Therefore, the groups of similarly sized tadpoles would consist—at least toward the end of the experiment—mostly of individuals above the predation risk threshold, while the variable groups of tadpoles with the same average mass include many smaller, predator‐vulnerable tadpoles. Indeed, in the absence of newts, 9.3% of surviving tadpoles were under 300 mg in the high size variation populations compared to 2.3% of tadpoles in the low size variation groups. Additionally, the presence of newts increased the average mass of tadpoles at the end of the experiment, likely due to selective predation upon smaller individuals (or, alternatively, via relaxed competition for resources). The presence of these smaller tadpoles appeared to produce a reduction in activity of tadpoles within the high variation group, which could mediate top‐down effects on the tadpole's dietary resources. Alternatively, or in addition to this mechanism, size variation *early* in the experiment could have resulted in the presence of larger, invulnerable tadpoles that do not respond behaviorally to the predators. These large, actively foraging tadpoles may stimulate movement in smaller conspecifics, exposing them to greater predation risk (Yamaguchi, Takatsu, & Kishida, 2016). We would expect this mechanism to have produced similar movement rates in the high variation and low variation populations exposed to predators, which we did not detect; however, this may have been a transitory effect before our observations began. Overall, the mechanisms by which predator presence differentially affects behavior and potentially survival in low versus high size variation populations need to be better elucidated. An important step would be conducting similar experiments to the one presented here, but with one or more of the following changes: (1) manipulating the mean size of the tadpoles to be above or below the threshold of predation, (2) use of both lethal and nonlethal (e.g., caged) predator treatments, separating the lethal effects of predation from changes in behavior, and (3) allowing and preventing interactions between conspecifics to determine the extent to which size classes directly alter each other's behavior.

However, these effects of size variation in the tadpoles should be temporally variable. Earlier in development, when the mean size of the tadpoles is below the size threshold at which they are no longer vulnerable, greater size variation would reduce the number of tadpoles in the population that are vulnerable to predators. As a consequence, a homogenous population of prey will contain a higher percentage of vulnerable individuals for a shorter period of time, while a variable population will have a smaller percentage of vulnerable prey present over a longer time frame. Over the entire course of development, similar total numbers of tadpoles may be consumed (provided there is no satiation of predators at high numbers of vulnerable prey), although the predator–prey dynamics may differ across points in development. As we sampled all data at one point in time, this present study can only evaluate instantaneous effects of size variation. It would be worthwhile to examine how these effects vary and accumulate over time, as the differences between size variation treatments may disappear, persist, or even become accentuated after all tadpoles grow beyond the predation risk size threshold.

The greater periphyton growth in mesocosms with both high size variation and newt presence is likely the result of the predator‐induced reduction in foraging effort (Carlson & Langkilde, 2014), an example of a behaviorally mediated trophic cascade (Schmitz, Beckerman, & O'Brien, 1997). Alternatively, newts feeding on many smaller tadpoles may increase nutrient availability in the water via excretion, and these nutrients may have stimulated periphyton growth (Costa & Vonesh, 2013). By either mechanism, the increased predation rate by newts upon high size variation tadpole populations yields enhanced periphyton production. Changes in patterns of newt predation on tadpoles may have also influenced zooplankton populations, either indirectly or directly. Feeding strikes by the newts increased in the presence of low size variation tadpole prey. This suggests greater effort expended toward foraging, a possible consequence of the reduced availability (due to size limitation) of high‐value food (tadpoles) leading to greater dependency on the far smaller alternative prey (zooplankton; Brophy, 1980). The lack of impact of tadpole size variation on change in newt mass suggests that they successfully compensated for the reduced tadpole prey availability by their increased foraging efforts and a possible dietary shift toward zooplankton. This should incur a cost for the newts in terms of greater energy expenditure, increased exposure to predators, and reduced time spent on other activities (e.g., seeking mates). Zooplankton abundance overall, and for all individual groups (except calanoid copepods), was not impacted by an interaction between newt presence and tadpole size variation. This is surprising, as greater foraging activity by newts upon zooplankton should have reduced the abundance of the latter. It is possible that the effects of increased foraging on microcrustaceans by newts may have been generally compensated for by other changes in the mesocosm community (e.g., greater foraging by tadpoles transporting nutrients from the periphyton to the water column, supporting greater phytoplankton communities as a resource for microcrustaceans; Wilbur, 1997), but this remains untested. The greater abundance of calanoid copepods in mesocosms containing highly size‐variable tadpoles with newts present suggests that the copepods could have been a favored alternative prey for the newts (hence their reduced abundance when low size variation tadpoles with few susceptible individuals were present) or that they responded favorably to increased nutrient availability from newt predation on high size variation groups of tadpoles. It is more difficult to explain the difference in calanoid abundance between size variation treatments in the absences of newts. This may be due to insufficiently studied mechanisms, such as predation by tadpoles upon microcrustaceans (Altig, Whiles, & Taylor, 2007; Schiesari et al., 2009), which may be impacted by size variation in the tadpoles. Further work is needed to elucidate the web of interactions between wood frog tadpoles, newts, and various microcrustacean taxa in order to fully understand how effects on the zooplankton community are mediated.

Together, these findings reveal that every component of this community we measured—tadpoles, their prey resources, their predators, and alternative prey for their predators—was impacted numerically or behaviorally by the extent of size variance in the tadpoles. This warrants that ecological studies pay increased attention toward considering size variability, and not only mean size, when characterizing populations, and suggests that functional diversity should be considered within species as well as between species. This study contributes to a recently growing body of evidence demonstrating that the focus on mean trait values in ecology ignores an important contributor to ecological interactions—the variance. Further empirical testing of how intraspecific trait variation influences communities (Bolnick et al., 2011) will allow us to better understand which mechanisms are most important and the extent of the impact. Size is a particularly amenable trait for such studies, as it is highly and often predictably variable, can be easy to measure and manipulate, and is an important determinant of an organisms niche (Woodward et al., 2005), as the results of our study show. Accounting for the role of intraspecific size variation, when measuring, manipulating, or modeling ecological processes, will allow us to expand our understanding of the role of biodiversity in ecosystems and refine our understanding of ecological interactions.

## AUTHOR CONTRIBUTIONS

BEC and TL conceived and designed the experiment. BEC performed the experiment and analyzed data. BEC and TL wrote the manuscript.

## CONFLICT OF INTEREST

None declared.
