# Supplementary figures and images for: Body size variation in aquatic consumers causes pervasive community effects, independent of mean body size
Source: Ecol Evol. 2017 Oct 22;7(23):9978–90. doi: 10.1002/ece3.3511 (PMC5723604; doi:10.1002/ece3.3511)

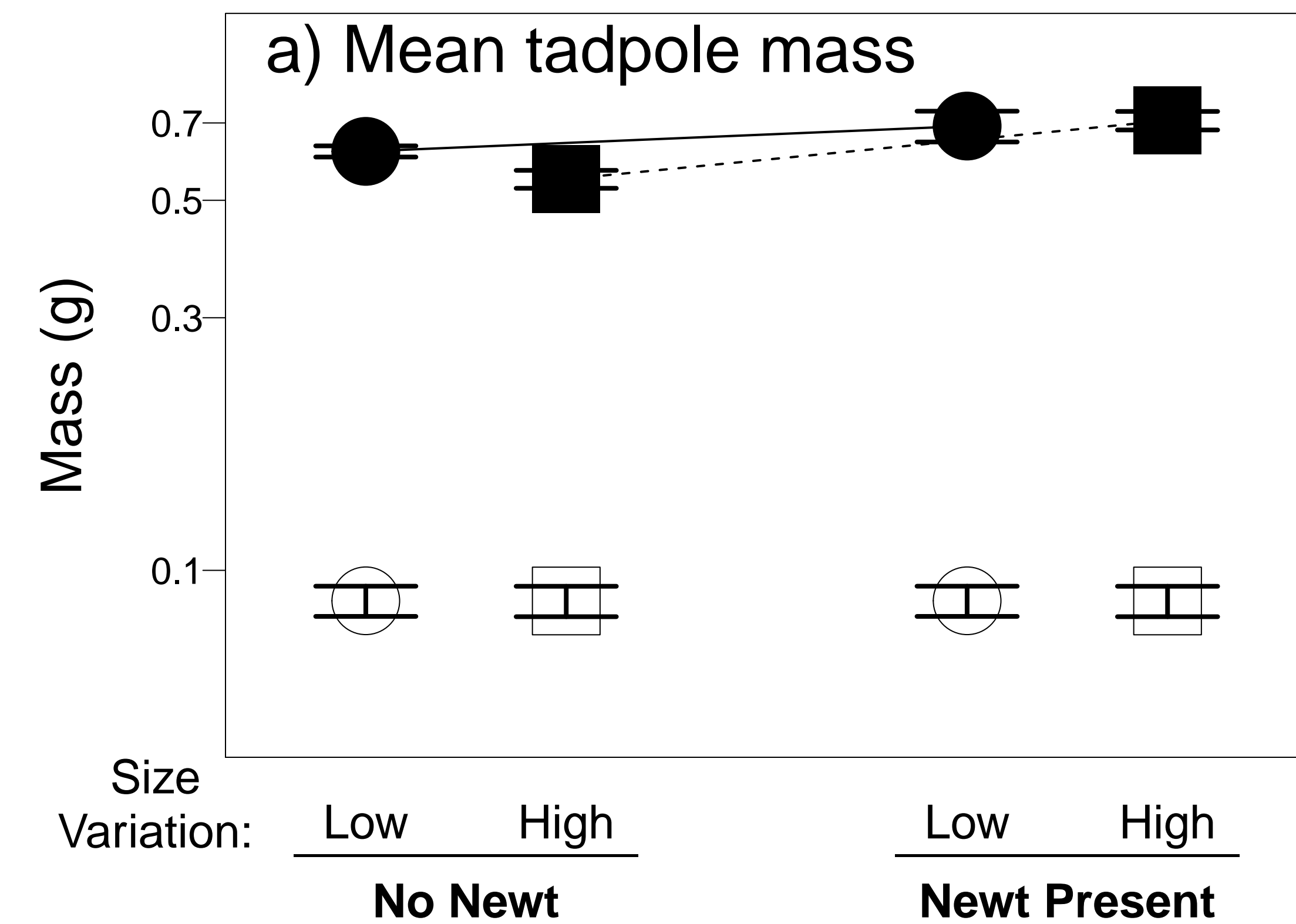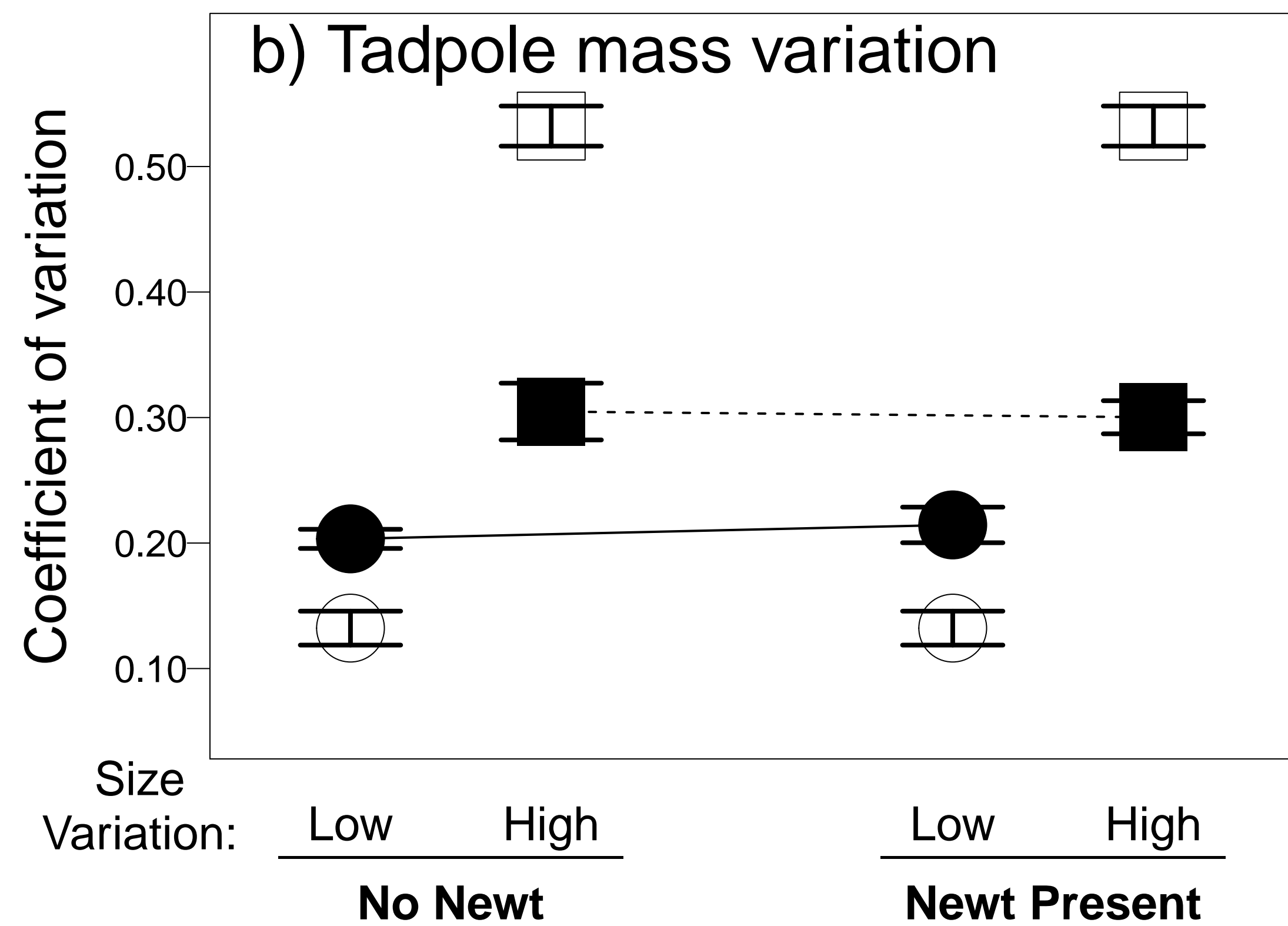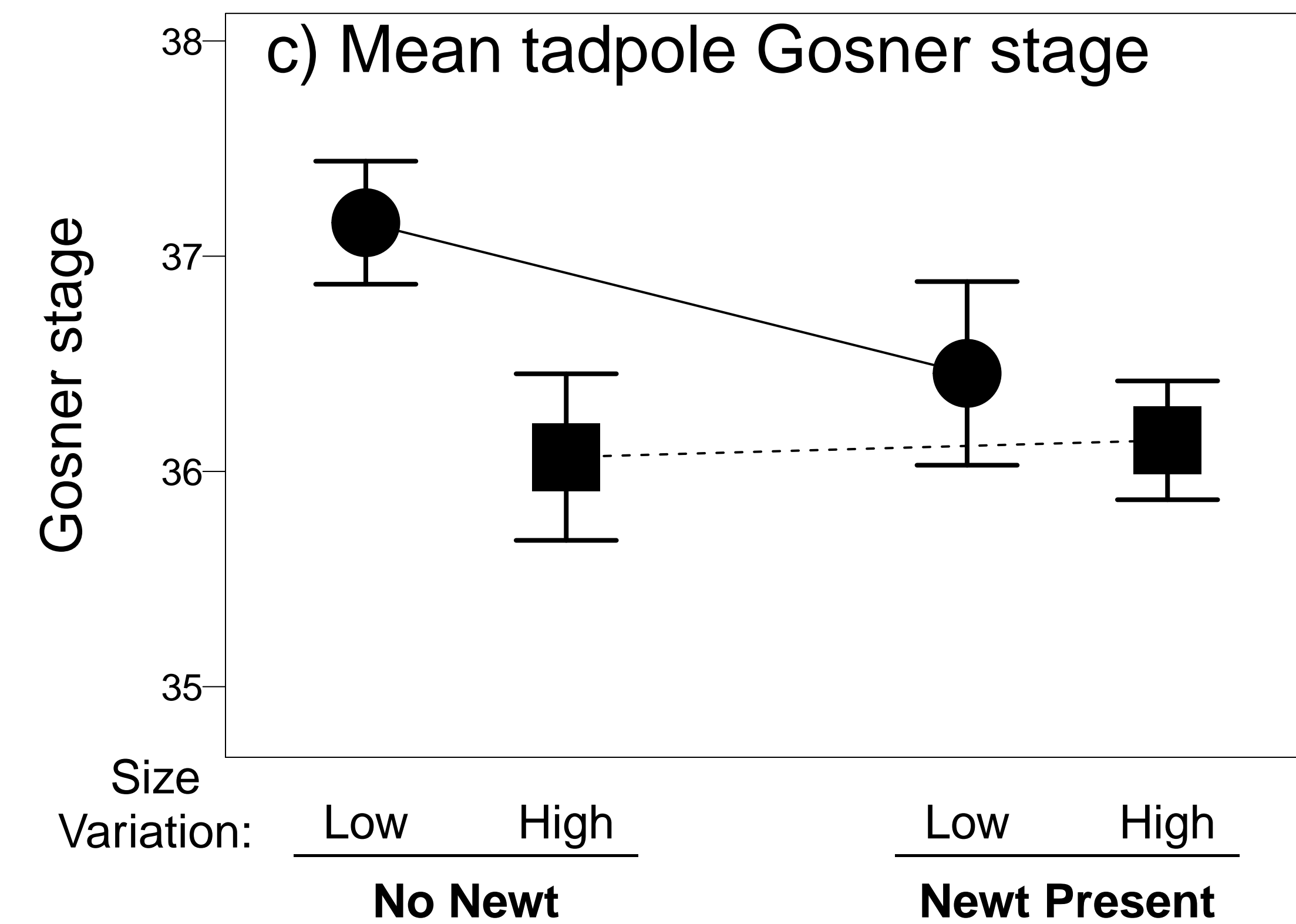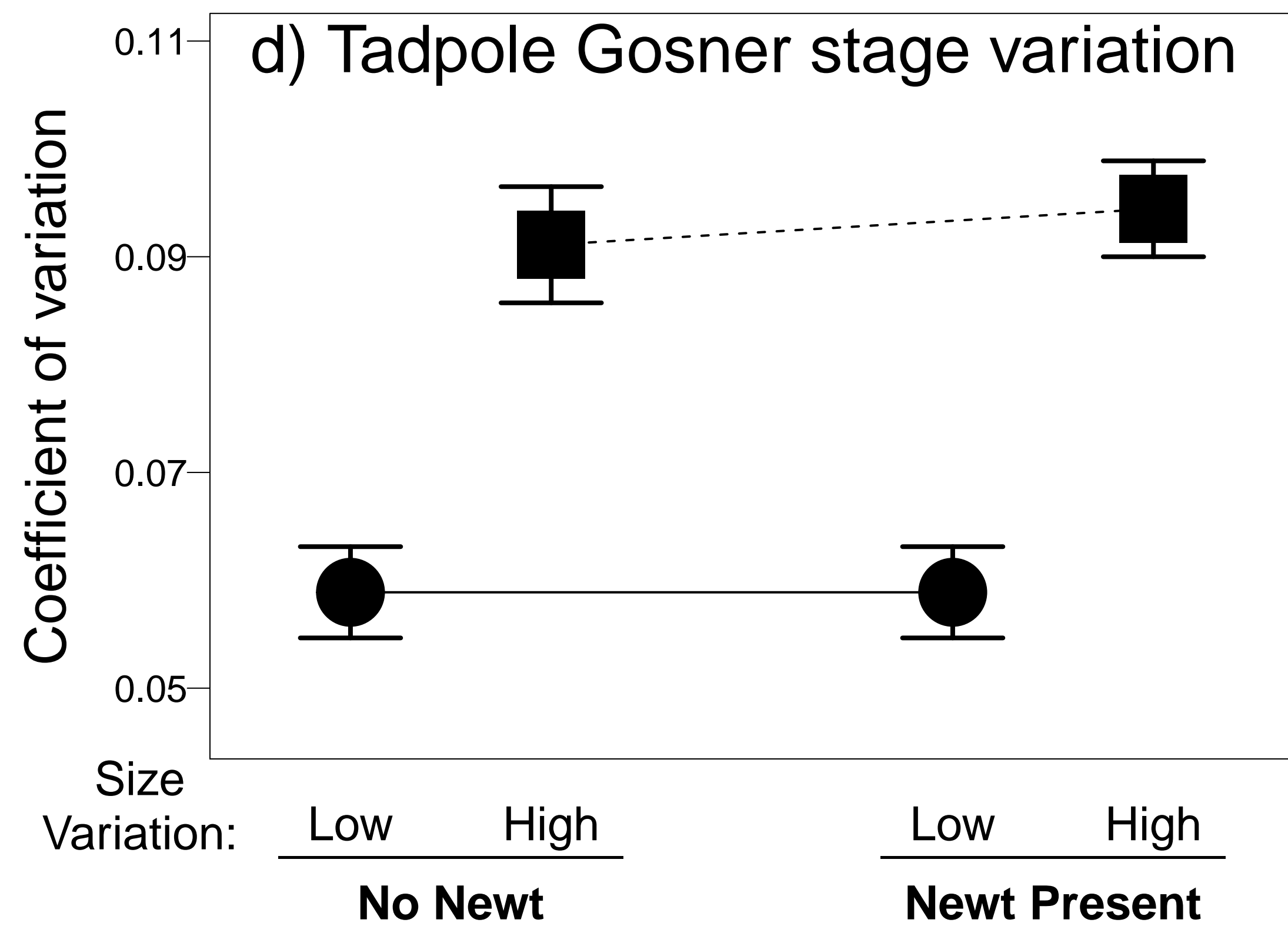

Supplement: Supplementary file 1 [file ECE3-7-9978-s001.pdf]

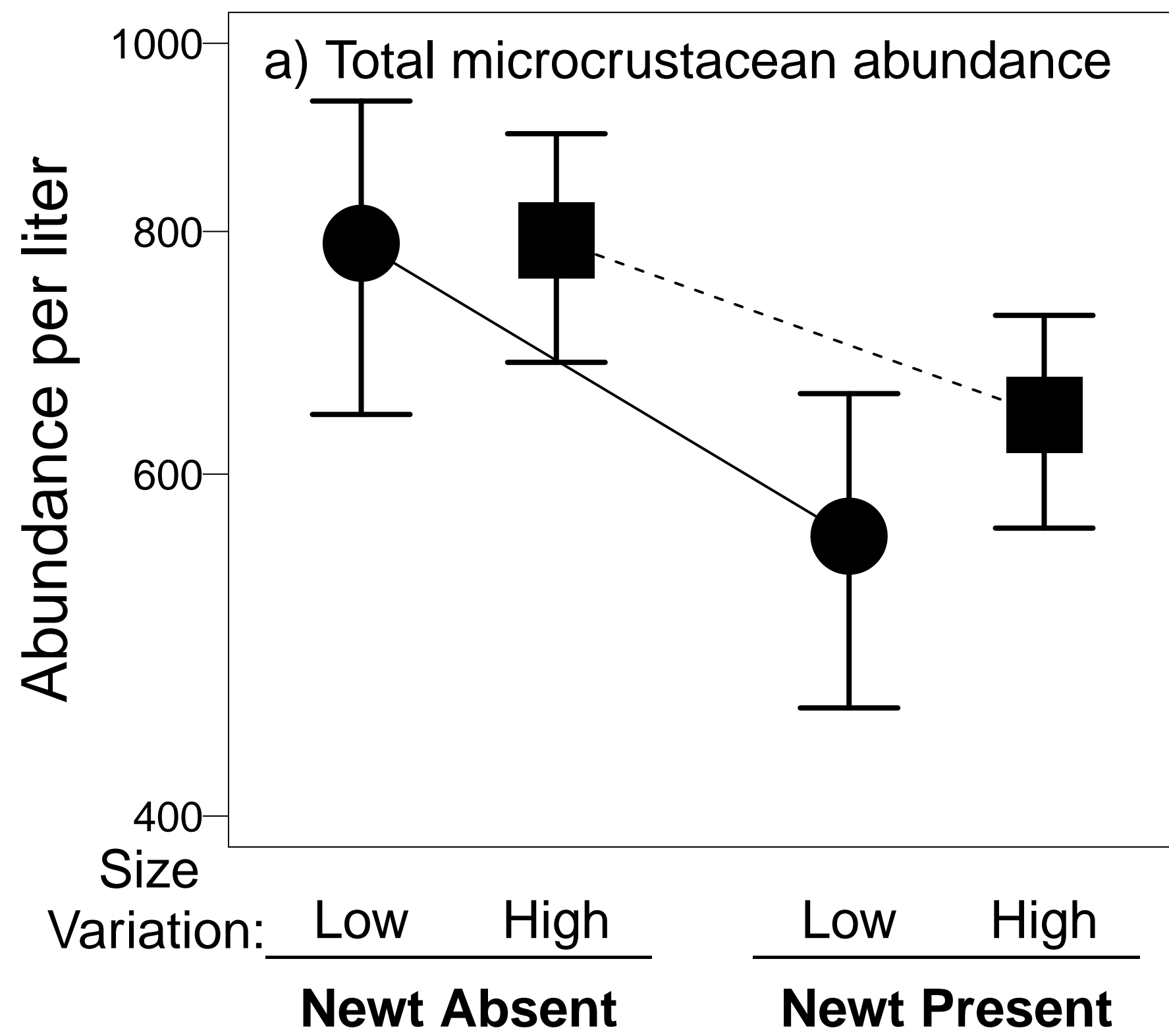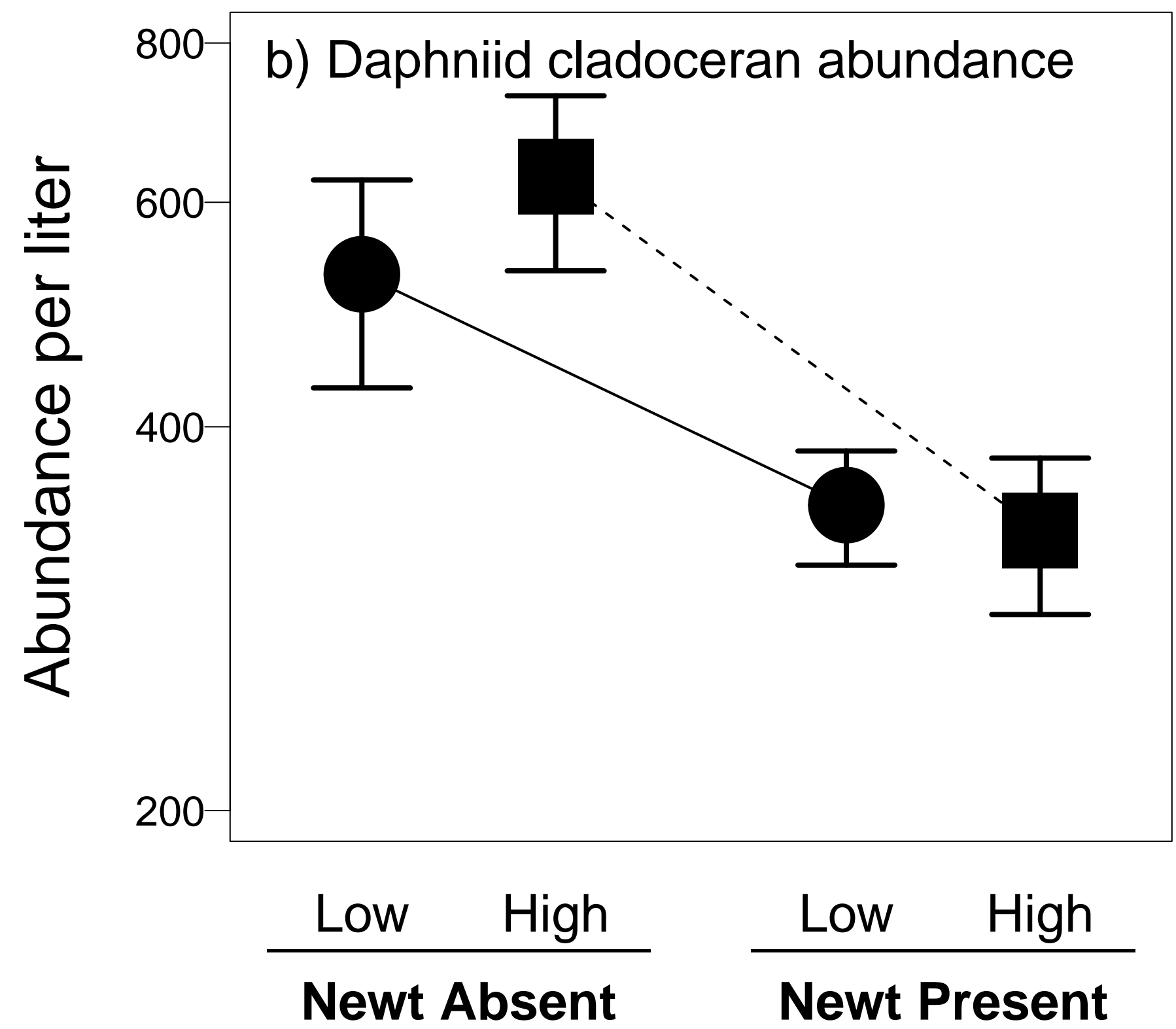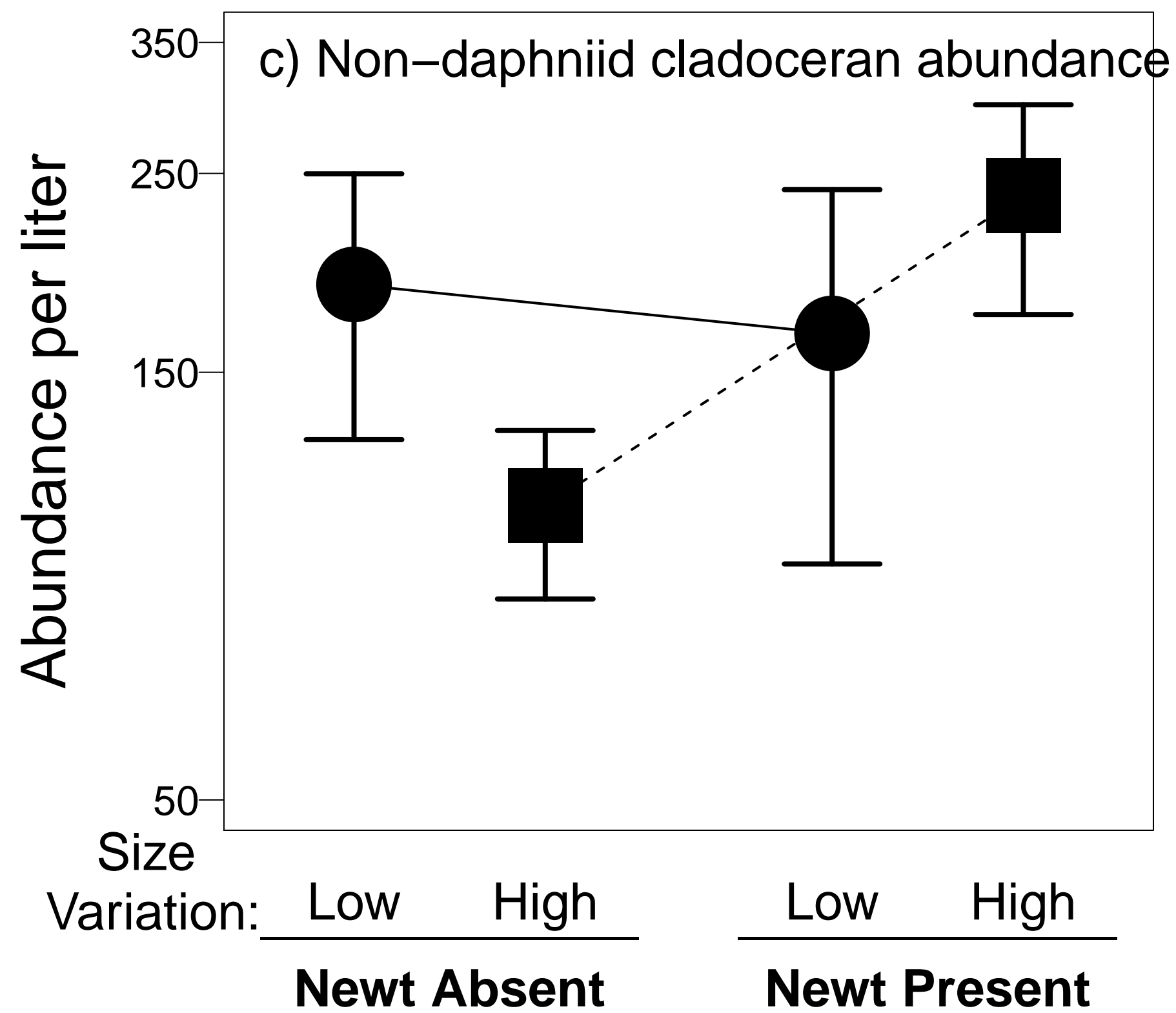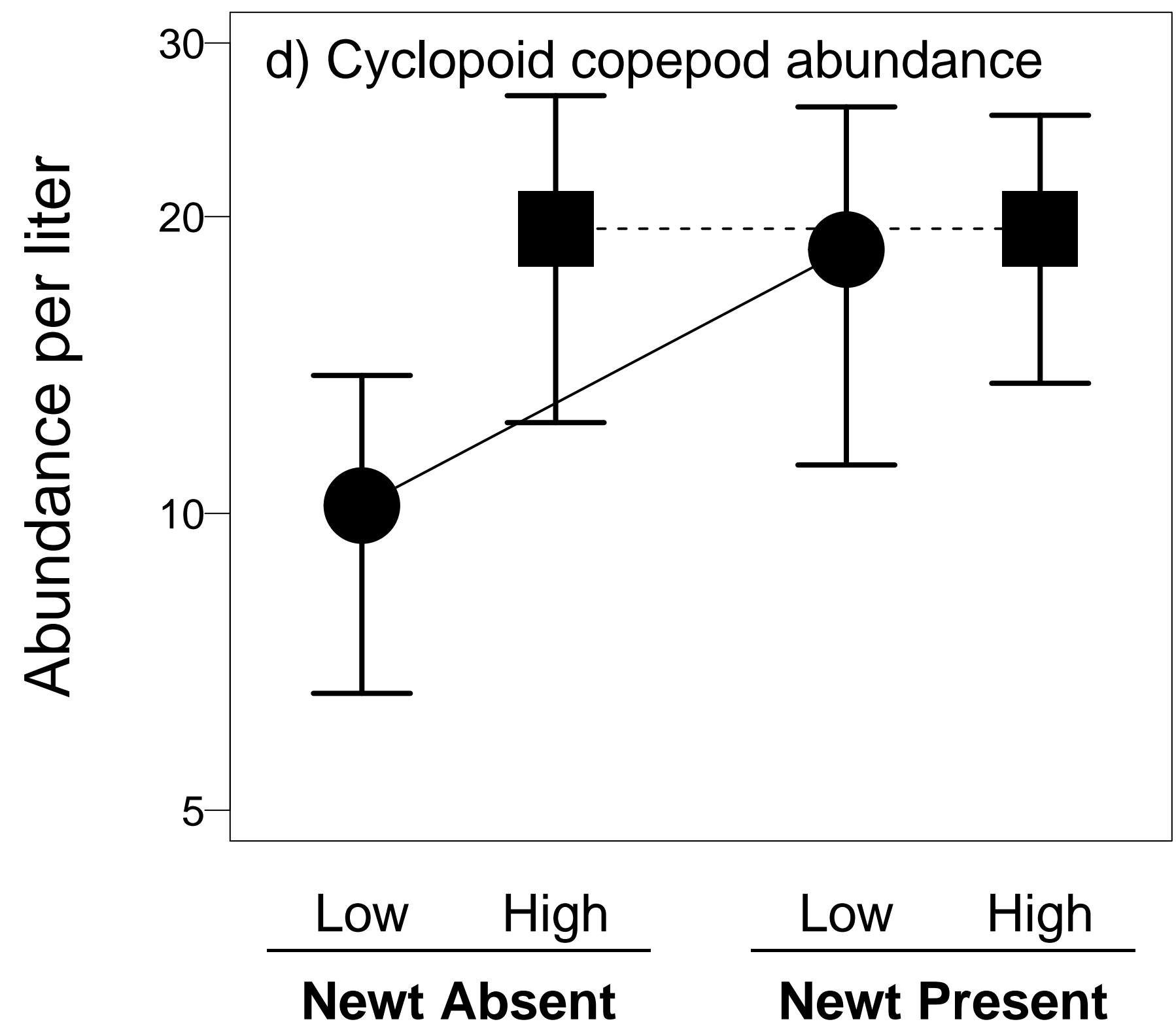

Supplement: Supplementary file 2 [file ECE3-7-9978-s002.pdf]
