# Supplementary material for: Body size variation in aquatic consumers causes pervasive community effects, independent of mean body size
Source: Ecol Evol. 2017 Oct 22;7(23):9978–90. doi: 10.1002/ece3.3511 (PMC5723604; doi:10.1002/ece3.3511)
Supplement: Supplementary file 3 [file ECE3-7-9978-s003.docx]

Supplementary Table A1. Effects of size variation and newt presence treatments and chosen covariates on tadpole behavior and log-transformed periphyton biomass. Analyses were performed using quasi-binomial GLMMs (a-b) or LMMs (c) with a random block effect (a-c) and a random mesocosm effect (a-b).

| *Response* | *Treatments/covariates* | *Test statistics* | *Significance* |
| --- | --- | --- | --- |
| (a) Proportion visible | Size variation | t_22_ = 1.02 | p = 0.32 |
|  | Newt present | t_22_ = -1.05 | p = 0.30 |
|  | Size × Newt | t_22_ = -1.18 | p = 0.25 |
|  | Mean tadpole mass | t_22_ = 0.77 | p = 0.45 |
|  | Mean tadpole stage | t_22_ = 1.44 | p = 0.16 |
|  |  |  |  |
| (b) Proportion active | Size variation | t_22_ = 0.14 | p = 0.89 |
|  | Newt present | t_22_ = 1.51 | p = 0.15 |
|  | Size × Newt | t_22_ = -2.17 | **p = 0.04** |
|  | Mean tadpole mass | t_22_ = -1.02 | p = 0.32 |
|  | Mean tadpole stage | t_22_ = -0.52 | p = 0.61 |
|  |  |  |  |
| (c) Periphyton biomass | Size variation | F_1, 24.5_ = 2.68 | p = 0.11 |
|  | Newt present | F_1, 23.5_ = 0.62 | p = 0.44 |
|  | Size × Newt | F_1, 20.9_ = 9.74 | **p = 0.005** |
|  | Mean tadpole mass | F_1, 25.2_ = 0.01 | p = 0.91 |
|  | Mean tadpole stage | F_1, 19.2_ = 0.36 | p = 0.56 |
|  | Mean tadpole visibility | F_1, 14.1_ = 5.73 | **p = 0.03** |
|  | Mean tadpole activity | F_1, 26.0_ = 2.91 | p = 0.10 |
|  | Tadpole survival | F_1, 26.0_ = 0.005 | p = 0.95 |
