# Supplementary material for: Body size variation in aquatic consumers causes pervasive community effects, independent of mean body size
Source: Ecol Evol. 2017 Oct 22;7(23):9978–90. doi: 10.1002/ece3.3511 (PMC5723604; doi:10.1002/ece3.3511)
Supplement: Supplementary file 4 [file ECE3-7-9978-s004.docx]

Supplementary Table A2. Effects of size variation and newt presence treatments and chosen covariates on microcrustacean abundance. Analyses were performed using quasi-Poisson GLMMs with a random block effect.

| *Response* | *Treatments/covariates* | *Test statistics* | *Significance* |
| --- | --- | --- | --- |
| (a) Total microcrustaceans | Size variation | t _19_ = -1.11 | p = 0.28 |
|  | Newt present | t _19_ = -0.74 | p = 0.47 |
|  | Size × Newt | t _19_ = 1.02 | p = 0.32 |
|  | Mean tadpole mass | t _19_ = -0.44 | p = 0.67 |
|  | Mean tadpole stage | t _19_ = -2.34 | **p = 0.03** |
|  | Mean tadpole visibility | t _19_ = -0.90 | p = 0.38 |
|  | Mean tadpole activity | t _19_ = -1.24 | p = 0.23 |
|  | Tadpole survival | t _19_ = 0.81 | p = 0.43 |
|  |  |  |  |
| (b) Daphniid cladocerans | Size variation | t _19_ = 0.19 | p = 0.85 |
|  | Newt present | t _19_ = -0.85 | p = 0.40 |
|  | Size × Newt | t _19_ = -0.35 | p = 0.73 |
|  | Mean tadpole mass | t _19_ = 0.15 | p = 0.88 |
|  | Mean tadpole stage | t _19_ = -1.85 | p = 0.08 |
|  | Mean tadpole visibility | t _19_ = -0.65 | p = 0.53 |
|  | Mean tadpole activity | t _19_ = -0.98 | p = 0.34 |
|  | Tadpole survival | t _19_ = 0.80 | p = 0.44 |
|  |  |  |  |
| (c) Non-daphniid cladocerans | Size variation | t _19_ = -2.32 | **p = 0.03** |
|  | Newt present | t _19_ = 0.08 | p = 0.94 |
|  | Size × Newt | t _19_ = 2.43 | **p = 0.03** |
|  | Mean tadpole mass | t _19_ = -0.49 | p = 0.63 |
|  | Mean tadpole stage | t _19_ = -2.01 | p = 0.06 |
|  | Mean tadpole visibility | t _19_ = -0.81 | p = 0.43 |
|  | Mean tadpole activity | t _19_ = -0.96 | p = 0.35 |
|  | Tadpole survival | t _19_ = 0.93 | p = 0.36 |
|  |  |  |  |
| (d) Calanoid copepods | Size variation | t _19_ = 1.05 | p = 0.31 |
|  | Newt present | t _19_ = 0.25 | p = 0.80 |
|  | Size × Newt | t _19_ = -0.79 | p = 0.44 |
|  | Mean tadpole mass | t _19_ = -1.41 | p = 0.17 |
|  | Mean tadpole stage | t _19_ = 1.86 | p = 0.08 |
|  | Mean tadpole visibility | t _19_ = -1.19 | p = 0.25 |
|  | Mean tadpole activity | t _19_ = 0.24 | p = 0.81 |
|  | Tadpole survival | t _19_ = -0.95 | p = 0.36 |
|  |  |  |  |
| (e) Cyclopoid copepods | Size variation | t _19_ = 1.05 | p = 0.31 |
|  | Newt present | t _19_ = 0.25 | p = 0.80 |
|  | Size × Newt | t _19_ = -0.79 | p = 0.44 |
|  | Mean tadpole mass | t _19_ = -1.41 | p = 0.17 |
|  | Mean tadpole stage | t _19_ = 1.86 | p = 0.08 |
|  | Mean tadpole visibility | t _19_ = -1.19 | p = 0.25 |
|  | Mean tadpole activity | t _19_ = 0.24 | p = 0.81 |
|  | Tadpole survival | t _19_ = -0.95 | p = 0.36 |
