# Supplementary material for: Body size variation in aquatic consumers causes pervasive community effects, independent of mean body size
Source: Ecol Evol. 2017 Oct 22;7(23):9978–90. doi: 10.1002/ece3.3511 (PMC5723604; doi:10.1002/ece3.3511)
Supplement: Supplementary file 5 [file ECE3-7-9978-s005.docx]

Supplementary Table A3. Effects of size variation and chosen covariates on newt mass change, movements, and feeding strikes. Analyses were performed using normally-distributed repeated measures ANOVA (a) or quasi-Poisson GLMMs (b,c) with random effects of block (a-c), mesocosm effects (a-c), and observation date (b,c).

| *Response* | *Treatments/covariates* | *Test statistics* | *Significance* |
| --- | --- | --- | --- |
| (a) Newt mass change | Size variation | F _1, 3_ = 0.01 | p = 0.94 |
|  | Mean tadpole mass | F _1, 3_ = 2.22 | p = 0.23 |
|  | Mean tadpole stage | F _1, 3_ = 0.44 | p = 0.56 |
|  | Mean tadpole visibility | F _1, 3_ = 0.01 | p = 0.94 |
|  | Mean tadpole activity | F _1, 3_ = 1.04 | p = 0.38 |
|  | Tadpole survival | F _1, 3_ = 0.15 | p = 0.72 |
| (b) Newt movements | Size variation | t _3_ = -2.87 | p = 0.06 |
|  | Mean tadpole mass | t _3_ = -2.93 | p = 0.06 |
|  | Mean tadpole stage | t _3_ = 1.21 | p = 0.31 |
|  | Mean tadpole visibility | t _3_ = 2.22 | p = 0.11 |
|  | Mean tadpole activity | t _3_ = -3.36 | **p = 0.04** |
|  | Tadpole survival | t _3_ = -1.74 | p = 0.18 |
| (c) Newt feeding strikes | Size variation | t _3_ = -3.06 | **p = 0.055** |
|  | Mean tadpole mass | t _3_ = -3.14 | **p = 0.052** |
|  | Mean tadpole stage | t _3_ = 2.73 | p = 0.07 |
|  | Mean tadpole visibility | t _3_ = 2.21 | p = 0.11 |
|  | Mean tadpole activity | t _3_ = -2.84 | p = 0.07 |
|  | Tadpole survival | t _3_ = -0.72 | p = 0.52 |
