# Supplementary material for: Body size variation in aquatic consumers causes pervasive community effects, independent of mean body size
Source: Ecol Evol. 2017 Oct 22;7(23):9978–90. doi: 10.1002/ece3.3511 (PMC5723604; doi:10.1002/ece3.3511)
Supplement: Supplementary file 6 [file ECE3-7-9978-s006.docx]

**Supplementary Material: Appendix S1 for: Carlson, B. E. and Langkilde, T. 2017. Body size variation in aquatic consumers causes pervasive community effects, independent of mean body size. – Ecology and Evolution.**

*Statistical analyses*

Generally, we analyzed all response variables as a 2 x 2 factorial experiment with main effects of tadpole size variation and presence/absence of newts and an interaction between size variation and predator presence. Block was included as a random intercept to account for minor differences among blocks in multiple variables. We used mixed effects ANOVAs (with random block intercepts), fit by least squares, to analyze treatment effects on the mean and CV of tadpole mass and Gosner stage at the conclusion of the experiment. For mean mass, variances were substantially unequal so we used a conservative significance level of p = 0.025 to correct for violation of model assumptions (Keppel and Wickens 2004). Because mean mass differed among blocks at the beginning of the experiment, we also analysed the effects of the treatments on the change in mean mass (final mean mass minus initial mean mass); this analysis yielded qualitatively similar results as the analysis using final mass only.

Tadpole survival was analyzed as a quasi-binomially-distributed response (proportion of surviving tadpoles) in a generalized linear mixed model (GLMM) fit using penalized quasi-likelihood (PQL) with fixed effects evaluated using the Wald t-test and random intercepts for each block (Bolker *et al*. 2009). A quasi-binomial distribution was used instead of a binomial distribution due to overdisperson violating model assumptions. We tested for treatment effects on behaviour by calculating tadpole activity rates (number of moving tadpoles/number of visible tadpoles) and tadpole visibility (number of visible tadpoles/number of tadpoles surviving to the end of the experiment). Both variables were treated as quasi-binomially-distributed in GLMMs, with random intercepts for block and mesocosm (nested within block) to account for repeated observations on each mesocosm (six observations per mesocosm). We also included observation date as a factor because exploratory analyses revealed substantial variation in tadpole behaviour among days; this is likely a result of differences in such variables as temperature and cloud cover that affect the behaviour and observability of the tadpoles. These models were again fit with PQL (Bolker *et al*. 2009). We also performed the same analyses but including mean mass and mean Gosner stage at the conclusion of the experiment as covariates. This was done to help elucidate the extent to which treatment effects on behaviour may have been mediated by their impacts on the average size and stage. We approach these particular models with caution, however, as any relationships between these covariates and the treatments could produce indirect (non-causal) relationships between response variables and mean size/stage or survival. Such collinearity between predictors in the models would reduce the power of significance tests, but we nonetheless present these results for full transparency.

Periphyton biomass was log-transformed to achieve normality. We analyzed periphyton biomass using mixed effects ANCOVAs (with random block intercepts), fit by least squares, to analyze treatment effects with a covariate indicating whether any larval chironomid fly tube casings were present on the tiles when periphyton was collected. Chironomid tubes occurred on the tiles from 15 of 36 mesocosms and were included in the total periphyton measurement, clearly increasing the mass of the periphyton on tiles where they occurred (t-test: t_30_ = 2.56, p = 0.02). We also evaluated a model with survival and mean size, Gosner stage, activity rate, and visibility as covariates to explore their role in mediating treatment effects. To test overall effects of treatments on the microcrustacean community, we first performed a mixed effects multivariate ANOVA (MANOVA) using log-transformed counts of each of the four identified microcrustacean groups as response variables. We followed with quasi-Poisson-distributed GLMMs (allowing for overdispersion) to conduct univariate tests with counts of each individual group of microcrustaceans and the total count of crustaceans as the response variables, fitting models with PQL (Bolker *et al*. 2009), again both with and without the covariates described above for periphyton.

Newt responses could only be considered in the “predator present” mesocosms, so in these cases treatment terms in the models were reduced to only a main effect of tadpole size variation. We calculated newt “growth” (mass gain) as the proportional change in mass from the beginning to the end of the experiment. This was analysed as mixed effects ANOVA, fit by least squares, with a random intercept for mesocosm number nested within blocks to reflect the non-independence of the two newts in each mesocosm. F-tests were used to evaluate effects in the model. The number of newt movements and lunges toward prey were analysed with a more complex random effects structure in which dates of observations were nested within mesocosm (which was nested within blocks) to account for repeated observations on each mesocosms (over six observation days) and observations of each of the two newts in every mesocosm on each day. For newt movements and lunges, we used a quasi-Poisson distribution (to account for overdispersion) and included an offset for the duration of time the newts were observed. These models were again fit with PQL and significance tested with Wald t-tests (Bolker et al. 2009). As before, we also examined models with the covariates relating to tadpole size, behaviour, and survival (described above) for all newt response variables.

**References:**

Bolker, B. M., Brooks, M. E., Clark, C. J., Geange, S. W., Poulsen, J. R., Stevens, M. H. H., & White, J.-S. S. (2009). Generalized linear mixed models: a practical guide for ecology and evolution. *Trends in Ecology & Evolution*, *24*, 127–135.

Keppel, G. & Wickens, T. D. (2004). *Design and analysis: a researcher’s handbook.* 4^th^ edition. Pearson.
